# Unravelling the theories of pre-eclampsia: are the protective pathways the new paradigm?

**DOI:** 10.1111/bph.12977

**Published:** 2015-02-27

**Authors:** Asif Ahmed, Wenda Ramma

**Affiliations:** 1 Vascular Therapeutics Unit, Aston Medical School, Aston UniversityBirmingham, UK; 2 Department of Pathology, Beth Israel Deaconess Medical Center, Harvard Medical SchoolBoston, MA, USA

## Abstract

Pre-eclampsia is a vascular disorder of pregnancy where anti-angiogenic factors, systemic inflammation and oxidative stress predominate, but none can claim to cause pre-eclampsia. This review provides an alternative to the ‘two-stage model’ of pre-eclampsia in which abnormal spiral arteries modification leads to placental hypoxia, oxidative stress and aberrant maternal systemic inflammation. Very high maternal soluble fms-like tyrosine kinase-1 (sFlt-1 also known as sVEGFR) and very low placenta growth factor (PlGF) are unique to pre-eclampsia; however, abnormal spiral arteries and excessive inflammation are also prevalent in other placental disorders. Metaphorically speaking, pregnancy can be viewed as a car with an accelerator and brakes, where inflammation, oxidative stress and an imbalance in the angiogenic milieu act as the ‘accelerator’. The ‘braking system’ includes the protective pathways of haem oxygenase 1 (also referred as Hmox1 or HO-1) and cystathionine-γ-lyase (also known as CSE or Cth), which generate carbon monoxide (CO) and hydrogen sulphide (H_2_S) respectively. The failure in these pathways (brakes) results in the pregnancy going out of control and the system crashing. Put simply, pre-eclampsia is an accelerator–brake defect disorder. CO and H_2_S hold great promise because of their unique ability to suppress the anti-angiogenic factors sFlt-1 and soluble endoglin as well as to promote PlGF and endothelial NOS activity. The key to finding a cure lies in the identification of cheap, safe and effective drugs that induce the braking system to keep the pregnancy vehicle on track past the finishing line.

**Linked Articles:**

This article is part of a themed section on Pharmacology of the Gasotransmitters. To view the other articles in this section visit http://dx.doi.org/10.1111/bph.2015.172.issue-6

## Tables of Links

**Table:** 

TARGETS
**Nuclear hormone receptors**^*a*^^2004^
PPAR-γ
**Catalytic receptors**^*b*^^2004^
VEGFR-1
VEGFR-2
**Enzymes**^*c*^^2004^
Akt (PKB)
Arginase
CSE
CBS
HO-1
HO-2
MPST
NOS1 (neuronal NOS)
NOS2 (inducible NOS)
NOS3 (endothelial NOS)

**Table:** 

LIGANDS	
Aspirin	IL-6
Asymmetric dimethylarginine (ADMA)	L-arginine
BH4	L-NAME
Bilirubin	Nitric oxide (NO)
Biliverdin	Pravastatin
Bradykinin	Sildenafil
cGMP	TNF-α
Cystathionine	TxA2
Cysteine	VCAM-1
DL-propargylglycine	VEGF-A
Endothelin-1 (ET-1)	Vitamin C
Fibronectin	Von Willebrand factor
Homocysteine	

These Tables list key protein targets and ligands in this article which are hyperlinked to corresponding entries in http://www.guidetopharmacology.org, the common portal for data from the IUPHAR/BPS Guide to PHARMACOLOGY (Pawson *et al*., 2014) and are permanently archived in the Concise Guide to PHARMACOLOGY 2013/14 (^*a,b,c*^Alexander *et al*., 2013a,b,c).

## Introduction

Pre-eclampsia is a major cause of maternal death worldwide (Lowe *et al*., 2009). It is characterized by the onset of new hypertension with proteinuria after 20 weeks of gestation. There are no effective pharmacological agents to treat pre-eclampsia. The only solution is the premature termination of the pregnancy. Although maternal symptoms appear to be largely resolved with the delivery of the baby, a growing body of evidence indicates that pre-eclampsia is associated with long-term health issues for both mother and baby (Smith *et al*., 2001; Bellamy *et al*., 2007; MacDonald *et al*., 2007). The human placenta is central to the development of pre-eclampsia. The incidence of pre-eclampsia increases as pregnancy proceeds from singleton to twin, triplets and quadruplets as the mass of placenta increases. Pre-eclampsia also occurs in molar pregnancies further indicating that it is the placenta and not the fetus that causes the condition. This condition is still being debated as a ‘disease of theories’ as we are entering into the second half of the second decade of the 21st century. In this review, we hope to shed new light and provide a clear steer where researchers need to focus if we are to find a cure for this elusive disorder in the first quarter of this century.

## Re-evaluation of existing theories of pre-eclampsia

Abnormal spiral artery remodelling was first postulated over five decades ago (Brosens, 1964; 1977) and has been accepted as the underlying cause of pre-eclampsia (Khong *et al*., 1986; Feinberg *et al*., 1991; Brosens *et al*., 2002; Burton *et al*., 2009). Referred to as the two-stage process (Redman, 1992; Redman and Sargent, 2005; Roberts and Hubel, 2009), for over two decades, most researchers have argued that the development of pre-eclampsia stems from abnormal spiral artery modification leading to placental hypoxia, increase in oxidative stress and aberrant maternal systemic inflammatory responses (Naljayan and Karumanchi, 2013).

Recent studies have shown that such defects are not specific to pre-eclampsia (Lyall *et al*., 2013), and are also associated with placental abruption, preterm premature rupture of membranes and intrauterine fetal death (Avagliano *et al*., 2011), indicating that abnormal spiral artery remodelling may be a common underlying contributing factor for abnormal placentation, but is not specific to pre-eclampsia. Additionally, the immune-deficient Rag2^−/−^/γ_c_^−/−^ double-knockout mice that lack spiral artery remodelling exhibit no pre–eclampsia-like phenotype. Burke *et al*. concluded that neither gestational hypertension nor deficient placental growth was an outcome of impaired spiral artery remodelling (Burke *et al*., 2010). Moreover, pre-eclampsia may occur in twin pregnancy despite normal uterine artery velocity waveform (Rizzo *et al*., 1993). Finally, a meta-analysis on the performance of first trimester uterine artery Doppler showed similar predictive accuracy for pre-eclampsia and other complications such as stillbirths and abruption (Velauthar *et al*., 2014). The test had high specificity, but low sensitivity, and this pattern of performance was noticed across various conditions including pre-eclampsia. This once again indicates that the shallow trophoblastic invasion in the spiral arteries resulting in progressive utero-placental ischaemia may relate to a more generalized remodelling pathology and is not the initiator of pre-eclampsia *per se* (Burke and Karumanchi, 2013).

The concept that abnormal spiral artery remodelling leading to hypoxia to support the two-stage hypothesis is further challenged by other sets of data. Huppertz and colleagues report that failure of endovascular trophoblast invasion does not lead to hypoxia. Rather, all measurements available to date point to increased oxygen levels within the placenta in patients with a failure of spiral artery transformation (Huppertz *et al*., 2014). Moreover, elevated soluble fms-like tyrosine kinase 1 (sVEGFR-1 also known as sFlt-1) from pre-eclamptic placenta is unlikely to be due to hypoxia *per se* as pre-eclamptic placenta continues to generate substantially higher levels of sVEGFR-1, even after 24 h of culture *ex vivo* under atmospheric condition as compared with normal placental explants under identical experimental conditions (Ahmad and Ahmed, 2004). If we accept that the clinical signs of pre-eclampsia are due, in large part, to elevated levels of sVEGFR-1, these findings challenge the long held belief that pre-eclampsia arises because of placental hypoxia. Based on these studies, we argue that the failed remodelling of maternal spiral arteries leading to hypoxia has been misconceived, for many years, as the cause of pre-eclampsia.

Another theory that has long been adopted without convincing clinical evidence is the idea that an elevation in maternal systemic inflammation is the cause of pre-eclampsia (Redman *et al*., 1999). Although studies in rodents support the systemic maternal inflammatory response and placental ischaemia hypothesis (LaMarca *et al*., 2007; Cotechini *et al*., 2014), this is not supported by human cytokine data. In human pregnancy, the elevation in pro-inflammatory status does not seem to precede the onset of pre-eclampsia (Djurovic *et al*., 2002; Kronborg *et al*., 2011; Carty *et al*., 2012). Furthermore, it is not associated with the severity of the disorder as there is no observed significant difference among serum TNF-α and IL-6 levels in patients with mild pre-eclampsia, severe pre-eclampsia and hemolysis, elevated liver enzymes, low platelets syndrome in pre-eclampsia (Ozler *et al*., 2012). Moreover, pregnant women with elevated systemic inflammation had increased IL-6 levels, but normal angiogenic status without exhibiting symptoms of hypertension or proteinurea (Ramma *et al*., 2012). More importantly, a prospective longitudinal study of 2600 women with singleton pregnancies who went on to develop pre-eclampsia using multiplex analysis of inflammatory markers and cytokines showed that there was no increase in any of the cytokines before the onset of pre-eclampsia (Carty *et al*., 2012). In addition, antenatal corticosteroid treatment demonstrated that inflammation is unlikely to be the major contributor to severe pre-eclampsia or useful for therapeutic targeting (Nayeri *et al*., 2014). These findings weaken the hypothesis that inflammation is the cause of pre-eclampsia.

## Is endothelial activation the central phenomenon?

Maternal endothelial dysfunction is the central phenomenon responsible for the clinical signs of pre-eclampsia. Soluble E-selectin, a marker of endothelial cell activation, was found to be significantly higher at 12–16 weeks gestation in women who subsequently developed pre-eclampsia (Carty *et al*., 2012). One of the theories postulated to be responsible for maternal endothelial dysfunction is the excessive production of free radicals (Wickens *et al*., 1981; Tsukatani, 1983; Roberts *et al*., 1989; Davidge, 1998; Hubel, 1999). This theory led obstetricians and scientists to advocate that maternal supplementation with low-dose aspirin or antioxidants could reduce oxidative stress (Wallenburg *et al*., 1986; Schiff *et al*., 1990; Kharb, 2000; Poston *et al*., 2006). These studies were undertaken without clear mechanistic evidence as daily intake of vitamin C, for just 6 weeks, increases the plasma level of 8-oxoadenine, a marker for DNA damage mediated by oxygen radicals (Podmore *et al*., 1998). Subsequent extensive systematic reviews with meta-analysis revealed that there is little evidence to support the administration of low-dose aspirin or vitamins C and E for either high- or low-risk women to prevent pre-eclampsia (Rossi and Mullin, 2011; Villa *et al*., 2013). There may be some benefit in the reduction of perinatal death and other adverse perinatal outcomes of low-dose aspirin initiated before 16 weeks of gestation (Roberge *et al*., 2013).

In the last decade, the hypothesis that pre-eclampsia arise because of the loss of VEGF activity as a result of the ‘increase in the levels of endogenous soluble (s)VEGFR-1 (also known as sFlt-1) that may antagonize the beneficial effects of VEGF’ (Ahmed, 1997), has been validated. A number of studies have consistently shown that the imbalance in anti-angiogenic factors is most strongly associated with the clinical signs of pre-eclampsia and disease severity (Chappell *et al*., 2002; 2013; Maynard *et al*., 2003; Ahmad and Ahmed, 2004; Buhimschi *et al*., 2006; Crispi *et al*., 2006; Levine *et al*., 2006; Venkatesha *et al*., 2006; Ramma *et al*., 2012). Anti-angiogenic factors, sVEGFR-1 and soluble endoglin (sEng, also known as CD105) are increased before the clinical onset of pre-eclampsia (Levine *et al*., 2006), and pregnant rodents exposed to high circulating levels of sVEGFR-1 illicit severe pre–eclampsia-like symptoms (Ramma and Ahmed, 2011; Rana *et al*., 2014).

## Metaphorical view of pre-eclampsia

Metaphorically speaking pregnancy can be seen as a car with an accelerator and brakes, where inflammation, oxidative stress and an imbalance in the angiogenic milieu act as ‘accelerators’. It is the failure in the braking systems, the endogenous protective pathway, that result in the ‘accelerator’ going out of control until the system crashes, manifesting itself as pre-eclampsia (http://youtube/vWUCWbKo1dE). The identification of the braking system and how to enhance the brakes when the system starts to fail can restore balance and possibly cure pre-eclampsia. The strategy to identify a cure for pre-eclampsia needs to be centred on identifying cytoprotective pathways, which reduce sVEGFR-1, sEng, oxidative stress and inflammation. The study of enzyme systems, which generate gasotransmitters, has the potential to lead to the discovery of effective treatments for pre-eclampsia. The trial that uses pravastatin to ameliorate early-onset pre-eclampsia (StAmP) is the first such approach underway in the UK; similarly other clinical trials are now taking place in the USA and Australia.

## Endogenous cytoprotective pathway

**Figure 1:**
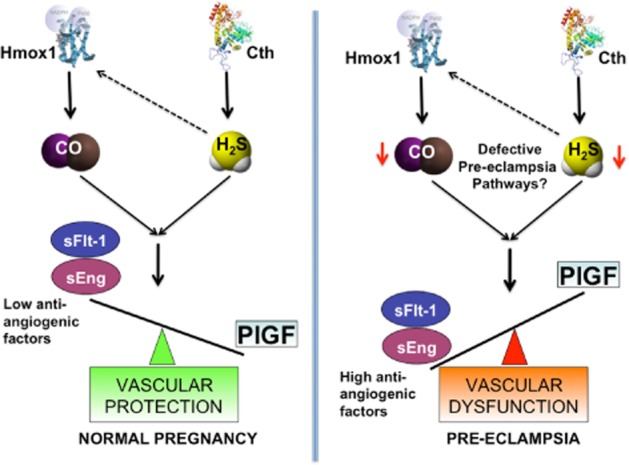
Effect of defective protective pathways in pre-eclampsia. This diagram illustrates that the defect in HO-1 (also referred to as Hmox) and CSE (also known as Cth), which generates signalling molecules, CO and H_2_S, and which results in an increase in sVEGFR-1 and sEng as well as a decrease in PlGF production. These are key factors responsible for vascular dysfunction in pre-eclampsia.

**Figure 2:**
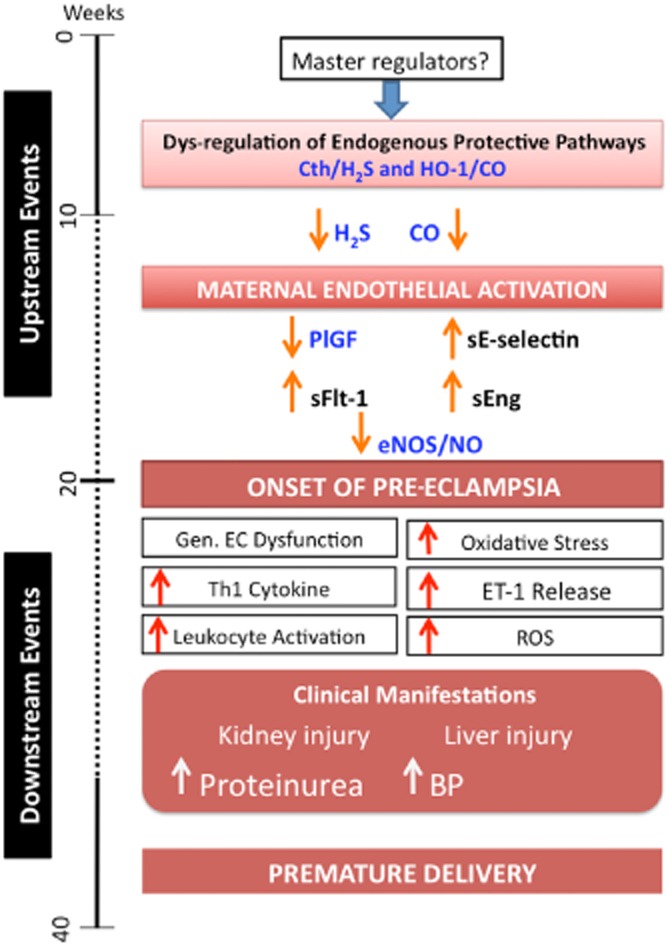
The accelerator and brake theory of pre-eclampsia. Schematic diagram to illustrate the sequence of events involved in the pathogenesis of pre-eclampsia. The upstream events consist of dysregulation of endogenous protective pathways – ‘the brakes’ – [CSE which generates H_2_S and HO-1 that produces CO] leading to maternal endothelial activation. As a consequence, there is an increase in anti-angiogenic factors – ‘the accelerator’ – (sVEGFR-1, sEng and soluble E-slectin and a decrease in angiogenic factors PlGF and eNOS, which generates NO). These biochemical changes lead to a generalized endothelial dysfunction, renal injury and generation of reactive oxygen species, which precedes the clinical onset of pre-eclampsia. After 20 weeks of gestation, the clinical symptoms manifest themselves as high BP and proteinurea, which are concurrent with excessive inflammation as indicated by increase in pro-inflammatory cytokines (Th1 cytokine production) and ET-1 release.

While most laboratories focused on finding the ‘accelerators’ of pre-eclampsia, our laboratory switched our efforts to identify key endogenous protective pathways; the control switch for the ‘accelerators’. The first ‘brake’ system identified was the haem oxygenase (HO)/carbon monoxide (CO) pathway (Ahmed *et al*., 2000). Recently, another ‘brake’ system discovered was the cystathionine-γ-lyase (CSE also known as Cth) (Wang *et al*., 2013), which produces hydrogen sulphide (H_2_S) (Bir and Kevil, 2013). These protective pathways inhibit sVEGFR-1 and sEng release (Cudmore *et al*., 2007; Wang *et al*., 2013) making them good candidates for the ‘brake’ theory outlined in Figures 1 and 2.

## Gasotransmitters

Gaseous signalling molecules, such as NO, CO and H_2_S, have all been hailed as promising molecules with clinical therapeutic potential largely because of their ability to act as vasodilators (Moncada, 1997; Dulak *et al*., 2008; Bir and Kevil, 2013). The enzyme systems that generate NO, CO and H_2_S are able to promote placental vasodilatation by regulating placental blood vessel tone *in vitro* and *in vivo* (Gude *et al*., 1990; Myatt *et al*., 1991; Gonzalez *et al*., 1995; Learmont and Poston, 1996; Odrcich *et al*., 1998; Ahmed *et al*., 2000; Bainbridge *et al*., 2002; Zhao *et al*., 2008; Patel *et al*., 2009; Holwerda *et al*., 2012; Cindrova-Davies *et al*., 2013; Wang *et al*., 2013). The discovery that NO, CO or H_2_S can promote placental blood flow is not surprising as these molecules have been shown to do this in a number of other vascular beds (Mustafa *et al*., 2009; Coletta *et al*., 2012). This property of these gaseous molecules by themselves is of limited therapeutic value in suppressing pre-eclampsia as illustrated by numerous NO studies (Johal *et al*., 2014). However, it is the additional properties of CO and H_2_S that makes them an attractive proposition in the search to identify therapies that would prevent pre-eclampsia.

## HO/CO system

HO is the rate-limiting enzyme responsible for the degradation of haem in the endoplasmic reticulum to generate equimolar amounts of biliverdin, free iron and CO (Tenhunen *et al*., 1969). Biliverdin is rapidly reduced to bilirubin, a potent antioxidant, by the cytosolic enzyme biliverdin reductase. CO is a potent vasodilator and also has anti-apoptotic properties. HO exists in two main isoforms, HO-1 and HO-2. The enzyme HO-2 is a 36 kDa protein that is constitutively expressed at high concentrations in the brain, testis and vascular endothelium. The inducible form, HO-1 is a 32 kDa protein that is widely distributed in the body, with high concentrations in the liver and the spleen. In mammalian tissues, HO-1 is induced by its substrate haem and also by heavy metals. Furthermore, stimuli that cause oxidative stress, such as peroxynitrite, modified lipids, hypoxia, hyperoxia, ischaemia/reperfusion, hyperthermia and endotoxic shock, up-regulate the expression of HO-1 (Sikorski *et al*., 2004). HO-1 via its products inhibits oxidative stress, inflammation and apoptosis (Dulak *et al*., 2008). A deficiency of HO-1 in humans results in severe and persistent endothelial damage as indicated by the marked elevation in thrombomodulin and von Willebrand factor (Yachie *et al*., 1999).

## HO/CO cytoprotective pathway in pregnancy

HO-1 and CO play a role in the maintenance of uterine quiescence during human pregnancy (Acevedo and Ahmed, 1998) and in modulating the utero-placental circulation (Ahmed *et al*., 2000; Lyall *et al*., 2000). More importantly, placental HO protected human placenta from cellular damage (Ahmed *et al*., 2000). A recent murine study showed that CO acts as a key molecule in successful pregnancy by modulating the uterine NK cells, which result in the promotion of the remodelling of maternal spiral arteries (Linzke *et al*., 2014). Decreased expression of HO has also been associated with human pregnancy disorders such as recurrent miscarriages (Denschlag *et al*., 2004), intrauterine growth retardation (Wong *et al*., 2012) and pre-eclampsia (Ahmed *et al*., 2000; Barber *et al*., 2001).

In pre-eclampsia, the functional importance of HO gained traction after the publication, which demonstrated that adenoviral HO-1 overexpression or CO exposure reduced sVEGFR-1 release from endothelial cells, while siRNA-mediated HO-1 knockdown increases sVEGFR-1 release (Cudmore *et al*., 2007). A small, but significant clinical study subsequently showed that the HO-1 mRNA was decreased in the chorionic villous (fetal placental cells) sampled at 11 weeks of gestation in women who went on to develop pre-eclampsia compared with normal pregnancies (Farina *et al*., 2008). Also, the addition of pre-eclampsia sera to the trophoblast increases sEng release and inhibits HO-1 expression (Aoki *et al*., 2012). Further support for HO-1 acting as a negative regulator of anti-angiogenic factors comes from a recent clinical study demonstrating that in samples of villous trophoblast obtained from women between 6 and 11 weeks of gestation undergoing elective abortion, the mRNA levels of HO-1 were significantly increased with gestational age, whereas the mRNA expression of sVEGFR-1 was significantly decreased with increasing gestational age (Miyagami *et al*., 2013). Finally, a recent study showed that the long allele of a guanine–thymine polymorphism in the HO-1 promoter region is associated with pre-eclampsia (Kaartokallio *et al*., 2014). Poor vascular compliance maybe associated with long allele. Collectively, these clinical studies reinforce our theory that placental HO-1 is (i) protective (Ahmed *et al*., 2000) and (ii) acts as a negative regulator of sVEGFR-1 and sEng (Cudmore *et al*., 2007). These findings support our proposed model of pre-eclampsia as an accelerator–brake defect disorder. Thus, we would argue that a partial loss of HO-1 activity early in gestation maybe responsible for the cascade of events that culminate in pre-eclampsia (Figure 2).

Interestingly another protective pathway appears to be mediated via the HO-1 system. Kenny's laboratory showed that the administration of a PPAR-γ agonist prevented the development of several of the pathophysiological characteristics associated with the reduced uterine perfusion pressure (RUPP) rat model of pre-eclampsia, via a HO-1-dependent pathway (McCarthy *et al*., 2011). Finally, the pharmacological induction of HO by cobalt protoporphyrin attenuates placental ischaemia-induced hypertension in this RUPP model (George *et al*., 2011) and the application of CO could normalize BP in gestational hypertensive HO-1^+/−^ pregnant mice (Linzke *et al*., 2014).

Another hallmark of pre-eclampsia is glomerular endotheliosis, which results in poor filtration and increased protein in the urine (Maynard *et al*., 2003). Apart from the administration of sVEGFR-1, VEGF neutralizing antibody to non-pregnant rats also results in glomerular endothelial cell damage and proteinuria (Sugimoto *et al*., 2003). Cancer patients receiving anti-VEGF therapy exhibit pre–eclampsia-like symptoms, suggesting that decreased bioavailability of VEGF causes these symptoms (Kabbinavar *et al*., 2003). Unfortunately, the effect of loss of HO-1 activity on kidney function in a pre-eclampsia setting is unknown and warrants investigation.

## CO paradox in pregnancy

The source of CO produced in the body, including in the human placental chorionic villi, was shown to originate from haem, primarily through the action of HO (Ahmed *et al*., 2005). Women with pre-eclampsia have a significantly reduced level of CO in their exhaled breath compared with those with healthy pregnancies indicating a decreased in HO activity (Baum *et al*., 2000; Kreiser *et al*., 2004). Interestingly, smoking during pregnancy reduces the incidence of pre-eclampsia, despite being associated with spontaneous abortion, stillbirth, preterm labour, fetal growth restriction and placental abruption (Conde-Agudelo *et al*., 1999). Smokers are also known to have reduced levels of circulating sVEGFR-1 (Levine *et al*., 2006). The smoking paradox was explained by the experimental observation that exposure to CO reduces endothelial and placental sVEGFR-1 and sEng release (Cudmore *et al*., 2007). Furthermore, cigarette smoke extract was shown to induce the expression of HO-1 in placental explants (Sidle *et al*., 2007) and decrease sVEGFR-1 release from placental villous explants without affecting the placental apoptotic status (Mehendale *et al*., 2007). Taken together, these studies showed that CO from cigarette smoke accounted for the reduced incidence of pre-eclampsia in smokers through the inhibition of sVEGFR-1 release (Cudmore *et al*., 2007; Farina *et al*., 2008; Zhao *et al*., 2009; Ahmed, 2011). The theory put forward in the May 2000 issue of Molecular Medicine demonstrating that HO offers protection against placental cytotoxic damage associated with pre-eclampsia appears to hold true, based on the supporting data, which has emerged over the last decade. HO-1 is one of the survival breaks against pre-eclampsia (Figure 2).

## CSE/H_2_S system

H_2_S is a gaseous signalling molecule, which promotes vasodilatation (Zhao *et al*., 2001), exhibits cytoprotective anti-inflammatory properties (Zanardo *et al*., 2006), protects against cellular damage induced by reperfusion injury (Elrod *et al*., 2007) or lethal hypoxia (Blackstone and Roth, 2007), and stimulates angiogenesis in the vasculature (Papapetropoulos *et al*., 2009). Endogenous H_2_S production is regulated by three enzymes CSE (also known as Cth), cystathionine-β-synthase (CBS) and 3-mercaptopyruvate sulfurtransferase (MPST), and generated from the substrates cystathionine, homocysteine, cysteine and mercaptopyruvate respectively (Bir and Kevil, 2013). CBS is most abundantly expressed in the brain (Kery *et al*., 1994) whereas CSE is the principal enzyme responsible for the endogenous production of H_2_S in the vasculature (Yang *et al*., 2008; Kabil *et al*., 2011). Administration of the CSE selective inhibitor (DL-propargylglycine, PAG) leads to elevated BP and vascular remodelling in the rat (Yan *et al*., 2004) and H_2_S levels are reduced in pulmonary hypertensive rats (Yanfei *et al*., 2006). Mice lacking CSE develop age-dependent hypertension, severe hyperhomocysteinaemia, and endothelial dysfunction (Yang *et al*., 2008).

## CSE/H_2_S pathway in pregnancy

The effects of low levels of H_2_S showed normal fetal growth and development during pregnancy and it increased delivery time in rats (Hayden *et al*., 1990) suggesting H_2_S may exhibit tocolytic characteristics. Indeed H_2_S inhibits spontaneous contractility in isolated pregnant rat uterine (Sidhu *et al*., 2001) and human myometrial strips (Hu *et al*., 2011; You *et al*., 2011; Robinson and Wray, 2012), similar to what was reported for CO (Acevedo and Ahmed, 1998). This is an area worthy of further investigation. Can myometrial-specific changes in HO-1 or CSE influence parturition?

An H_2_S-producing system exists within the utero-placental unit (Patel *et al*., 2009; You *et al*., 2011). For almost 10 years it has been know that both H_2_S and CO act as vasodilators (Leffler *et al*., 2006). It is therefore reassuring that perfusion of normal placenta with an H_2_S donor results in vasorelaxation of preconstricted vasculature (Cindrova-Davies *et al*., 2013). H_2_S may act as a vasodilator in the placental circulation as is the case for H_2_S in a number of other vascular beds.

Recent immunohistochemical localization studies with CSE and CBS reported contradictory findings on placental pathologies. Holwerda and colleagues observed no changes in CSE expression, but a decrease in CBS expression in placenta from severe pre-eclampsia (Holwerda *et al*., 2012). In contrast, CSE immunoreactivity was reduced in placenta from pregnancies with severe early-onset growth-restriction and pre-eclampsia (Cindrova-Davies *et al*., 2013; Wang *et al*., 2013). Real-time PCR confirmed reduced CSE mRNA in pre-eclamptic women and it was associated with decreased levels of plasma H_2_S (Wang *et al*., 2013). However, these observational studies provide little insight into the contributing role of this enzyme system or its gaseous product in pre-eclampsia.

Given that the placenta is a highly vascular organ, Wang *et al*. proposed that the dys-regulation of the CSE/H_2_S pathway would promote placental abnormalities and contribute to a pre–eclampsia-like condition. Like the HO-1 system, which negatively regulates sVEGFR-1 and sEng (Cudmore *et al*., 2007), the CSE pathway has been shown to have similar capabilities. Endothelial CSE knockdown by siRNA increased the endogenous release of sVEGFR-1 and sEng while adenoviral-mediated CSE overexpression inhibited their release from endothelial cells. Furthermore, inhibition of CSE activity by administration of PAG to pregnant mice induced hypertension, liver damage, elevated sVEGFR-1 and sEng and promoted abnormal labyrinth vascularization in the placenta and decreased fetal growth. These symptoms were reversed when the inhibitor was supplemented with GYY4137, a slow-releasing H_2_S-generating compound demonstrating that the effect of CSE inhibitor was due to inhibition of H_2_S production (Wang *et al*., 2013). These findings imply that endogenous H_2_S is required for healthy placental vasculature and a decrease in CSE/H_2_S activity may contribute to the pathogenesis of pre-eclampsia. Indeed, H_2_S was also reported to protect against acute myocardial ischaemia/reperfusion injury. Treatment with H_2_S donor (SG-1002) offers cardioprotection via up-regulation of the VEGF–Akt–NOS3–NO pathway (Kondo *et al*., 2013).

The importance of H_2_S in preventing damage is further supported by a recent study showing that H_2_S attenuates adenovirus-mediated overexpression of sVEGFR-1-induced hypertension and renal damage in non-pregnant Sprague Dawley rats (Holwerda *et al*., 2014). This study also showed that rat renal VEGF-A mRNA expression increased significantly with intraperitoneal injection of H_2_S donor, sodium hydrosulphide (NaHS) treatment and NaHS stimulated podocytes in culture to release VEGF (Holwerda *et al*., 2014). H2S is known to stimulate VEGF; exposure of vascular smooth muscle cells to H_2_S up-regulates hypoxia inducible factor (HIF)-1α and VEGF protein levels and increased HIF-1α binding activity under hypoxic condition (Liu *et al*., 2010). Recently, VEGFR-2 was reported as the direct target of H_2_S and a VEGF receptor inhibitor suppressed angiogenesis induced by H_2_S (Tao *et al*., 2013). These findings indicate that H_2_S promotes angiogenesis via VEGF receptor activation.

Clearly, CSE may also play a key role in placental development as inhibition of CSE activity by PAG inhibited trophoblast invasion *in vitro* (Wang *et al*., 2013). Inhibition of CSE activity in early (first trimester) human placental explants obtained from termination of pregnancy results in a marked reduction in placenta growth factor (PlGF) production (Wang *et al*., 2013). In pre-eclampsia, the maternal circulating level of PlGF is decreased well before the onset of the symptoms (Levine *et al*., 2004; 2006). Although the significance of this decrease in PlGF remains to be explained in women, studies by Kumasawa *et al*. and colleagues show that administration of PlGF in lentiviral sVEGFR-1-infected mice depresses the level of sVEGFR-1 and ameliorated hypertension, glomerular endotheliosis and proteinuria in the mice (Kumasawa *et al*., 2011). The loss of activity in the H_2_S-producing enzyme CSE may account for the reduction in PlGF in pre-eclampsia (Wang *et al*., 2013). As PlGF is one of the earliest maternal circulating markers to be reduced in women destined to develop early-onset pre-eclampsia (Chappell *et al*., 2002; 2013), CSE/H_2_S activity may be upstream to PlGF and could be an earlier biomarker as well as a key regulator that keeps the level of PlGF sufficiently high to counteract the deleterious effect of sVEGFR-1, which can induce pre–eclampsia-like symptoms if allowed to go unchecked.

Pharmacological studies using a PAG inhibitor have their limitations because of off-target effects and have been shown to inhibit other pyridoxal-5′-phosphate-dependent enzymes (Whiteman *et al*., 2011); the lack of selectivity for specific isozymes means future studies need to be carried out using gene inactivation approaches by delineating CSE in the placenta and maternal endothelium (Bir and Kevil, 2013).

## NOS3/NO system

NO is synthesized from the nonessential amino acid L-arginine (L-arg) by the enzyme NOS. There are three isoforms of NOS: NOS1 (neuronal), NOS2 (inducible) and NOS3 (endothelial). NOS1 and NOS3 are considered constitutive (cNOS). The NO produced in endothelial cells induces vascular smooth muscle relaxation via a cGMP-dependent pathway to promote vasodilatation. Other beneficial effects of NO in the vascular system include inhibition of ET-1 and TxA_2_ production, platelet aggregation, leukocyte adhesion and smooth muscle proliferation (Lowe, 2000). NO also inhibits production of VCAM-1 and fibronectin as well as low-density lipoprotein oxidation (Ahmed *et al*., 2009) and is critical for neovascularization (Bussolati *et al*., 2001; Ahmad *et al*., 2006).

Loss of endothelial NOS (NOS3) activity is an established contributor to endothelial dysfunction (Heitzer *et al*., 2001). NO is highly reactive as it has an unpaired electron, thus a dynamic competition between superoxide and lipid radicals for reaction with NO is inevitable. NO only stimulates superoxide-dependent lipid oxidation when the production rate of NO is less than superoxide (Rubbo *et al*., 2000). In endothelial cells, NOS3 exists in a homodimeric complex that is stabilized by the cofactor BH4. Decreased availability of BH4 results in ‘uncoupling’ of NOS3 activity and increases the production of superoxide (d'Uscio *et al*., 2001; Bendall *et al*., 2005).

## NOS3/NO pathway in pregnancy

Enzyme expression and localization studies by themselves can be misleading. However, blocking the production of NO by administering a NOS-inhibiting agent produces virtually all the symptoms of pre-eclampsia in pregnant mice and rats suggesting that the NOS pathway is a key player in this disorder (Lowe, 2000). A meta-analysis showed that genetic variations in the NOS3 gene contribute to an increased risk for pre-eclampsia (Dai *et al*., 2013). Indeed BH4 doubled NOS3 activity in a concentration-dependent manner in homogenates of first trimester and term placenta (Kukor *et al*., 2000) and uncoupled NOS3 and oxidative stress in a rat model of pregnancy-induced hypertension (Mitchell *et al*., 2007). However, BH4 concentrations in pre-eclamptic placenta were reported to be comparable with those of normal placenta (Kukor *et al*., 2000). In non-pregnant mice lacking NOS3, the sVEGFR1-induced pre-eclampsia phenotype in mice is aggravated (Li *et al*., 2012).

A nested case control study of screening for pre-eclampsia revealed that asymmetric dimethylarginine (ADMA), arginine and homoarginine at 11–13 weeks' gestation did not change in women who went on to develop late-onset pre-eclampsia, but L-arg and homoarginine levels were decreased in those who developed early-onset pre-eclampsia (Khalil *et al*., 2013). Interestingly, maternal serum ADMA concentration tends to increase during normal pregnancy, but ADMA concentrations in the second trimester were significantly elevated in pregnancies that later developed pre-eclampsia (Rizos *et al*., 2012).

An important cause of impaired endothelial NO production is the reduced availability of the NOS3 substrate L-arg. A study reported that plasma nitrite and superoxide dismutase activity were significantly lower while arginase activity and plasma endothelin levels were significantly higher in pre-eclamptic women than in normotensive pregnant women (Bernardi *et al*., 2008). Why arginase activity is increased during pregnancy is unknown, but should be a subject of future investigation. The Davidge laboratory have shown that an increased arginase expression in pre-eclampsia can induce the uncoupling of NOS as a source of superoxide in the maternal vasculature in pre-eclampsia, and suggested that L-arg supplementation in the face of oxidative stress could lead to a further increase in peroxynitrite (Sankaralingam *et al*., 2010). Thus, this brings into question L-arg supplementation as therapy without clear mechanistic evidence.

Maximum activity of NOS3 is achieved when the residue S1177, which is targeted by Akt, is phosphorylated and residue T497 is dephosphorylated. The constitutively active NOS3 mutant, S1177D enhances NO-mediated relaxation of bradykinin-stimulated porcine coronary arteries (Teupe *et al*., 2002). Basal production of NO from cells is markedly reduced in cells transfected with T497A and T497A/S1177D (TASD) NOS3 because of the generation of more superoxide anion that interact with NO. In contrast, NO production is augmented in cells transfected with S1177D NOS3 (Lin *et al*., 2003). Using adenoviruses expressing wild-type NOS3 and S1177D and TASD NOS3 mutants, we discovered that neither the constitutively active NOS3 mutant S1177D nor the TASD NOS3 mutants suppress VEGF-induced sVEGFR-1 release from endothelial cells. These unpublished findings are in marked contrast to studies in which sildenafil citrate reduces the plasma levels of sVEGFR-1 and sEng in L–NAME-treated Sprague Dawley pregnant rats (Ramesar *et al*., 2011). Sildenafil may exert its effect indirectly. Finally, storkhead box 1 (STOX1) overexpression switches the free radical balance from reactive oxygen species to reactive nitrogen species in the placenta (Doridot *et al*., 2014), which may deprive maternal NO and compromise blood flow or increase endothelial dysfunction. Could this act as a genetic switch altering vascular function and inhibiting the protective genes such as HO-1?

## Conclusion

Women destined to develop early-onset pre-eclampsia could be offered therapies that up-regulate ‘the endogenous cytoprotective pathway’. A cheap and widely available therapy against pre-eclampsia may be on the horizon if pravastatin (3-hydroxy-3-methylglutaryl-coenzyme A reductase inhibitors) proves to be ‘safe’ in randomized controlled trial (RCT) (StAmP). Although statins are contraindicated in pregnancy, the finding that statins inhibited sVEGFR-1 led to the UK regulatory authorities approving the first RCT on statins in pre-eclampsia. Today, there are two additional trials underway in Australia and the United States.

In order to develop additional therapies to tackle pre-eclampsia, basic research is urgently needed to elucidate fully the role of HO-1 and CSE and their metabolites. Murine models do not equate to pre-eclampsia in women, but they can replicate many of the pre-eclampsia symptoms. This offers very useful tools to perform proof of principle experiments to determine the role of specific genes and potential new therapies. H_2_S or CO may modulate NOS3 activity to prevent pre-eclampsia. It is established that one of the beneficial effects of statins in the prevention of cardiovascular events is their ability to enhance endothelial function by increasing NOS3 expression and activity (Xenos *et al*., 2005). The success of the StAmP trial may not only depend on its ability to up-regulate HO or inhibit sVEGFR-1, but on the power of statins to inhibit an array of stress factors associated with pre-eclampsia.

This review suggests new investigations with new thinking which challenges the existing dogma, employs the use of sophisticated inducible- and tissue-specific knockdown tools in what we refer to ‘the maternal and fetal vascular complications mouse models of pre-eclampsia’ combined with human clinical-based sample studies. For example, transcription factor STOX1 overexpressing pregnant mice exhibit symptoms of severe pre-eclampsia: gestational hypertension, proteinuria, and elevated plasma levels of sVEGFR-1 (soluble fms-like tyrosine kinase 1) and sEng (Doridot *et al*., 2013). We propose a new paradigm as outlined here in ‘the accelerator and brake’ hypothesis, which can encompass novel factors (Figure 2). We need to design rigorous tests for a given hypothesis and bring in a team-based approach, which can utilize resources from a range of settings. Studies need to encompass clinical samples, cell culture studies and proof of concept mouse models as outlined earlier. The criticism of mouse models in pre-eclampsia needs to stop, provided the animal data are backed up by human data as illustrated in a number of comprehensive studies (Maynard *et al*., 2003; Wang *et al*., 2013). Animal studies in pre-eclampsia should be accepted as powerful scientific tools where specific genes can be delineated in a tissue-specific fashion to address specific questions. We propose this approach for the cytoprotective pathway gasotransmitter enzymes, which we believe holds the greatest promise in the clinic and maybe to the underlining mechanism that is defective in pre-eclampsia. In conclusion, our discovery of the two gasotranmitter enzyme systems as protective pathways to treat or prevent preeclampsia (Figure 1) and the accelerator-brake model of preeclampsia is being used to develop new therapies, which aim to enhance the brakes when the system starts to fail. The StAmP trial is the first such approach to be completed. The future personalized pre-eclampsia therapy is looking promising!

## Abbreviations

ADMA: asymmetric dimethylarginine
BH4: tetrahydrobiopterin
CBS: cystathionine-β-synthase
CO: carbon monoxide
CSE (also known as Cth): cystathionine-γ-lyase
H_2_S: hydrogen sulphide
HO (also know as Hmox): haem oxygenase
L-arg: L-arginine
PAG: DL-propargylglycine
PlGF: placenta growth factor
RUPP: reduced uterine perfusion pressure
sEng: soluble endoglin
sVEGFR-1 (also known as sFlt-1: soluble fms-like tyrosine kinase-1
StAmP: pravastatin to ameliorate early-onset pre-eclampsia
